# Supplementary material for: Metabolite Fraction Libraries for Quantitative NMR Metabolomics
Source: Anal Chem. 2026 Jul 1;98(27):20255–64. doi: 10.1021/acs.analchem.6c01279 (PMC13373914; doi:10.1021/acs.analchem.6c01279)
Supplement: Supplementary file 1 [file ac6c01279_si_001.pdf]

Supplementary Information:
Metabolite Fraction Libraries for Quantitative NMR Metabolomics

Christopher Esselman[1,4], Kara Garrison[2,4], Leandro Ponce[3,4], Ricardo M. Borges[5], Frank Delaglio[6], *Arthur S. Edison[1,2,4]

1. Institute of Bioinformatics, University of Georgia, Athens, Georgia, USA 30602.
2. Department of Biochemistry and Molecular Biology, University of Georgia, Athens, Georgia, USA 30602.
3. College of Engineering, University of Georgia, Athens, Georgia, USA 30602.
4. Complex Carbohydrate Research Center, University of Georgia, Athens, Georgia, USA 30602.
5. Instituto de Pesquisa de Produtos Naturais Walter Mors, Universidade Federal do Rio de Janeiro, Rio de Janeiro, Brazil 21941902
6. Institute for Bioscience and Biotechnology Research, National Institute of Standards and Technology and the University of Maryland, Rockville, Maryland, USA 20850.

***Author Address**

Complex Carbohydrate Research Center, University of Georgia

315 Riverbend Road, Athens, GA, 30602-4712 USA

Email: aedison@uga.edu

SI Contents

Page number	Item
S2	**Figure Supplementary 1:** Experimental Workflow.
S3	**Figure Supplementary 2:** Details of metabolite fraction library peaks from SAND.
S4	**Figure Supplementary 3:** Fraction library of ground-truth mixture.
S5	**Figure Supplementary 4:** Demonstration of degraded galactose in ground-truth mixture.
S6	**Figure Supplementary 5:** Demonstration of oxidized cysteine in ground-truth mixture.
S7	**Figure Supplementary 6:** BATMAN fit of the ground-truth dataset using the full metabolite basis set (mBS).
S8	**Figure Supplementary 7:** BATMAN fit of the ground-truth dataset using the mBS with top 20 missing.
S9	**Figure Supplementary 8:** BATMAN fit of *N. crassa* mixture using the ground-truth mBS.
S10-S11	Ground-truth dataset methods.

Additional excel file: mFL_Supplementary_Tables.xlsx
1) Table 1. *N. crassa* Database Match
2) Table 2. Ground-Truth mBS
3) Table 3. mBS Concentrations from *N. crassa*
4) Table 4. Ground-Truth Mixtures

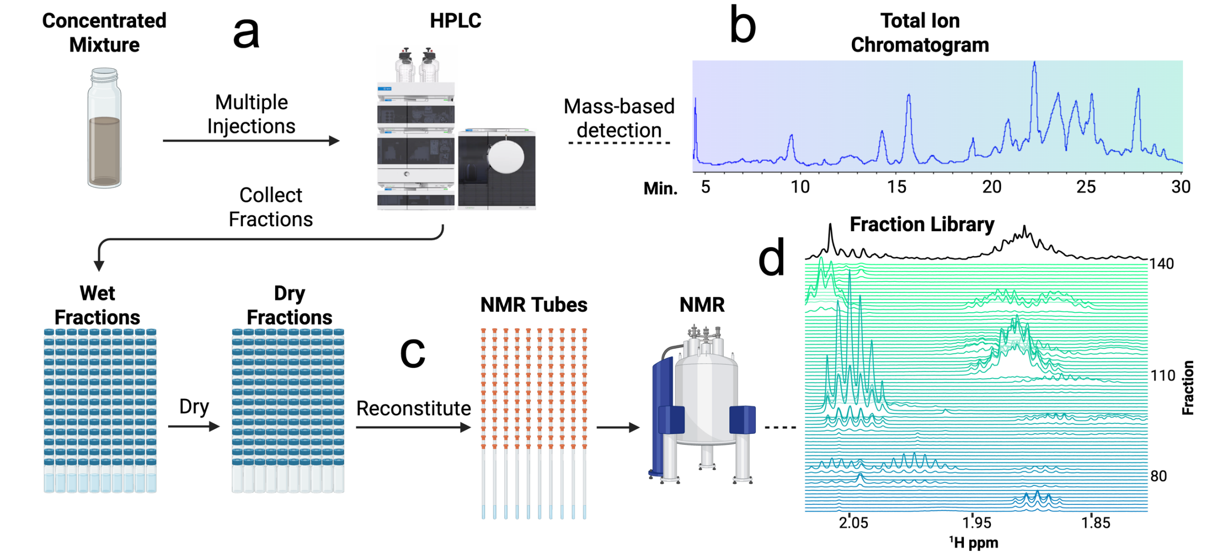

Figure Supplementary 1: Experimental workflow of creating a metabolite Fraction Library (mFL). **a**, A concentrated biological mixture is injected multiple times into an HPLC using the same fraction vials for each injection. **b**, If a mass spectrometer is coupled to the HPLC, a total ion chromatogram can be collected during fractionation. **c**, The fractions are then dried using a vacuum concentrator and reconstituted into NMR tubes. **d**, One-dimensional (1D) proton (^{1}H) NMR is collected for each fraction.

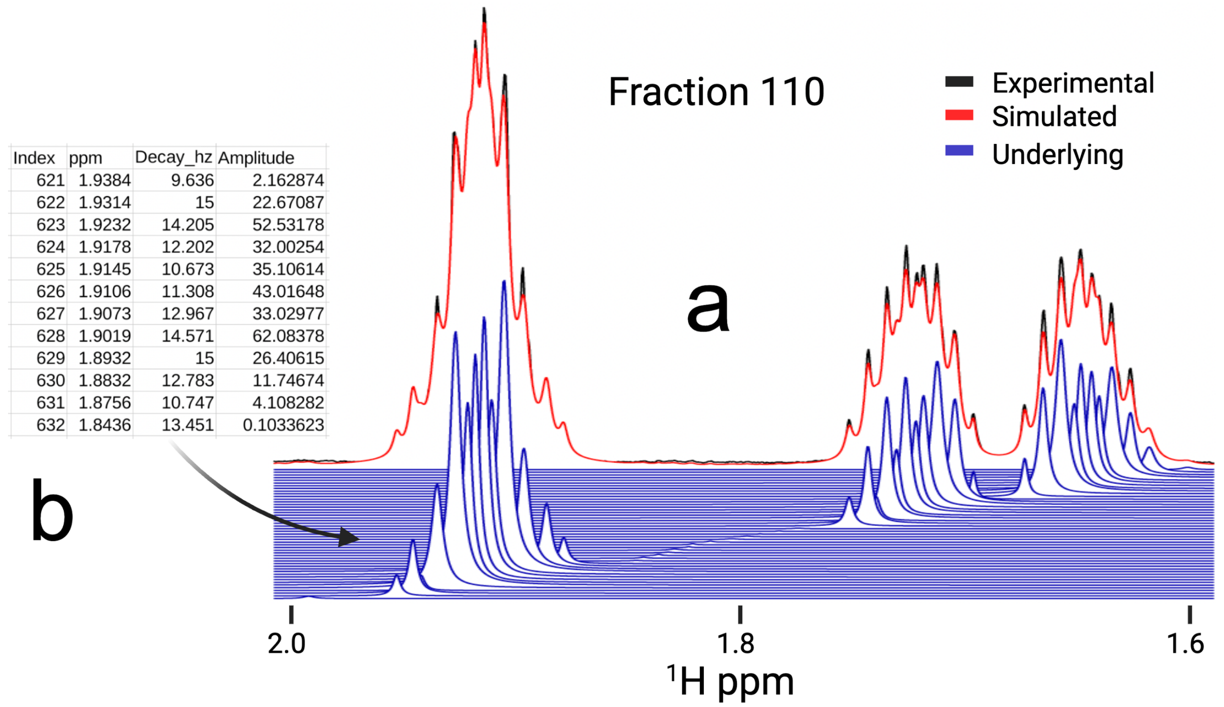

Figure Supplementary 2: Time domain modeling of NMR data via spectral automated NMR decomposition (SAND). **a**, The black spectrum is the original Fourier transformed (FT) data. The blue peaks are the frequency reconstructed underlying Lorentzian peaks time domain modeled by SAND. The red spectrum is the summation of the blue Lorentzian peaks. **b**, The SAND tabular domain data includes the frequency, decay, amplitude, and phase of every peak in the modeled spectrum. The phase is usually adjusted to zero before SAND.

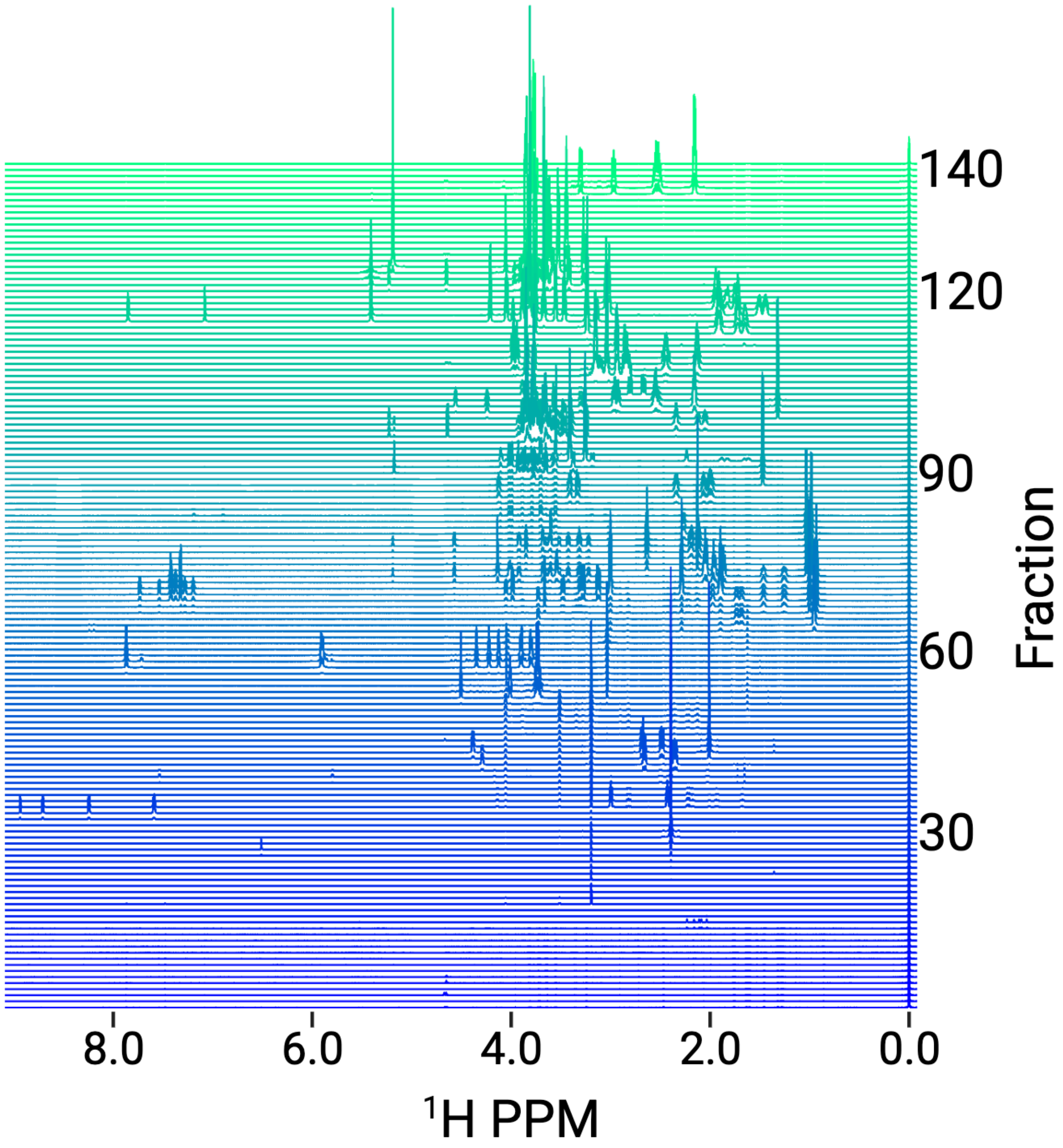

Figure Supplementary 3: Metabolite fraction library (mFL) of ground-truth mixture.

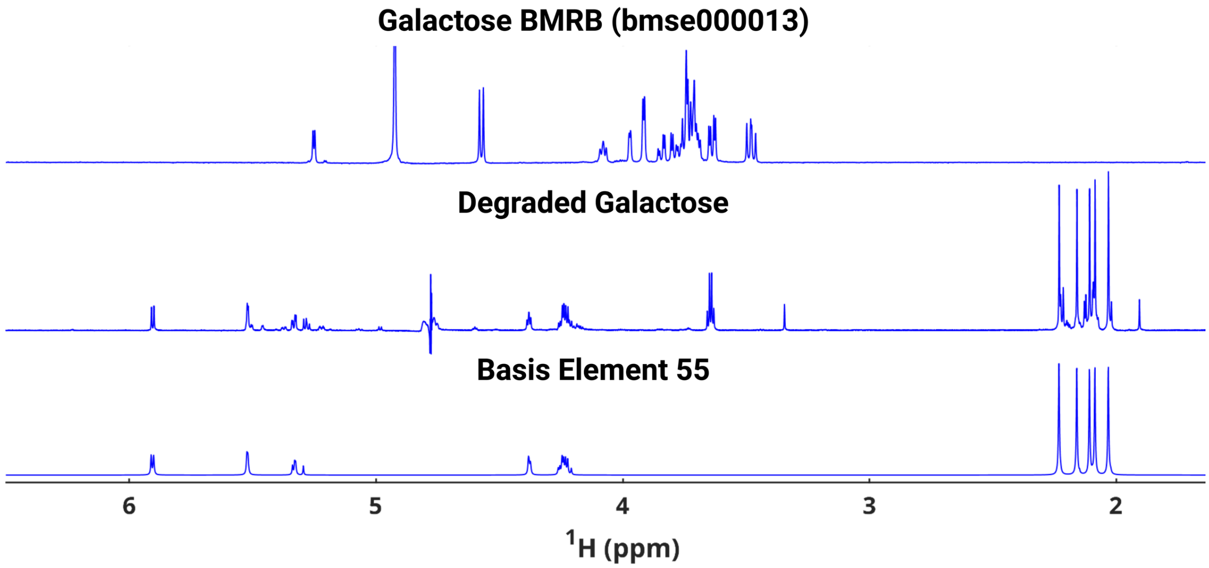

Figure Supplementary 4: BMRB reference spectrum (bmse000013) of galactose, the ground-truth spectrum of the degraded galactose sample, and metabolite basis element 55. Unexpectedly, the galactose obtained for this study does not match the known spectrum of galactose from the BMRB database. The peaks in the galactose sample that are not captured in basis element 55 may be peaks of another contaminant.

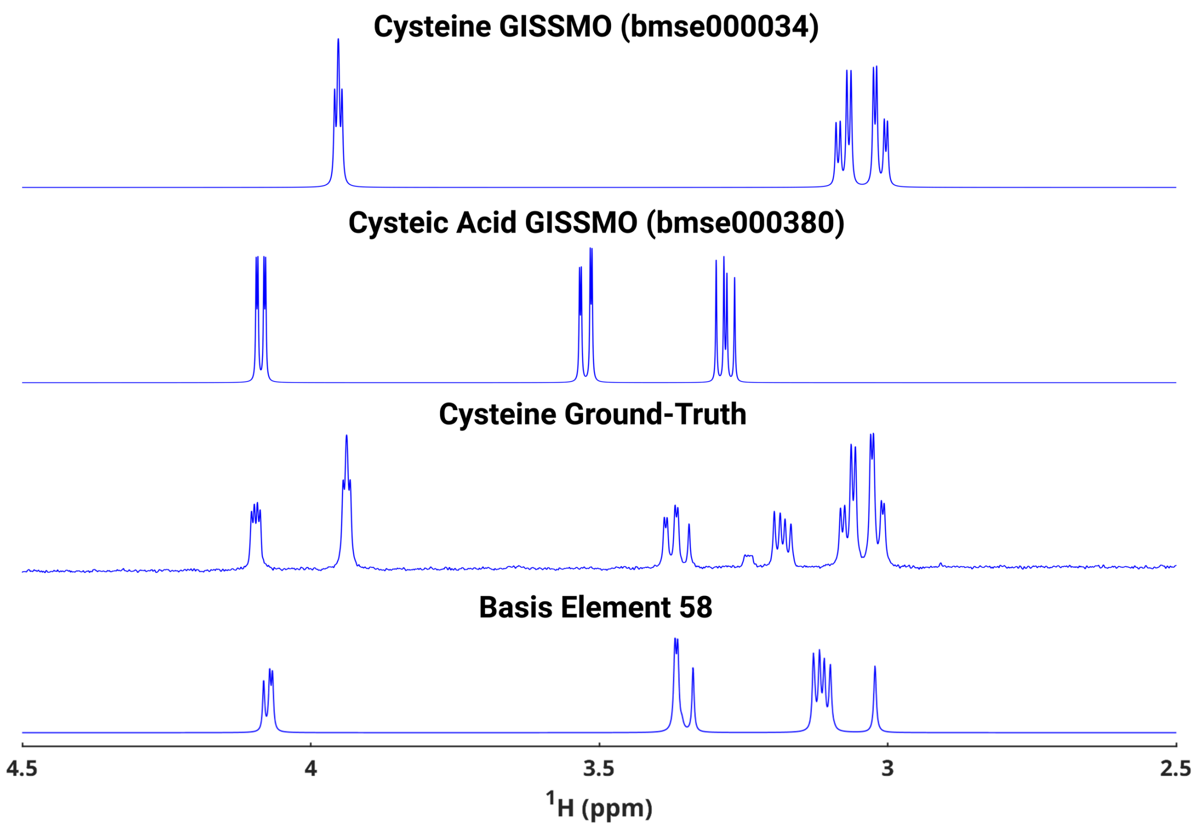

Figure Supplementary 5: GISSMO reference spectrum (bmse000034) of cysteine, GISSMO reference spectrum (bmse000380) of cysteic acid, the ground-truth spectrum of the cysteine sample, and metabolite basis element 58. The cysteine ground-truth sample exhibits resonances that resemble those of cysteine and its oxidation product, cysteic acid. Changes in chemical shift are likely due to variations in pH. We quantified cysteic acid resonances in basis element 58.

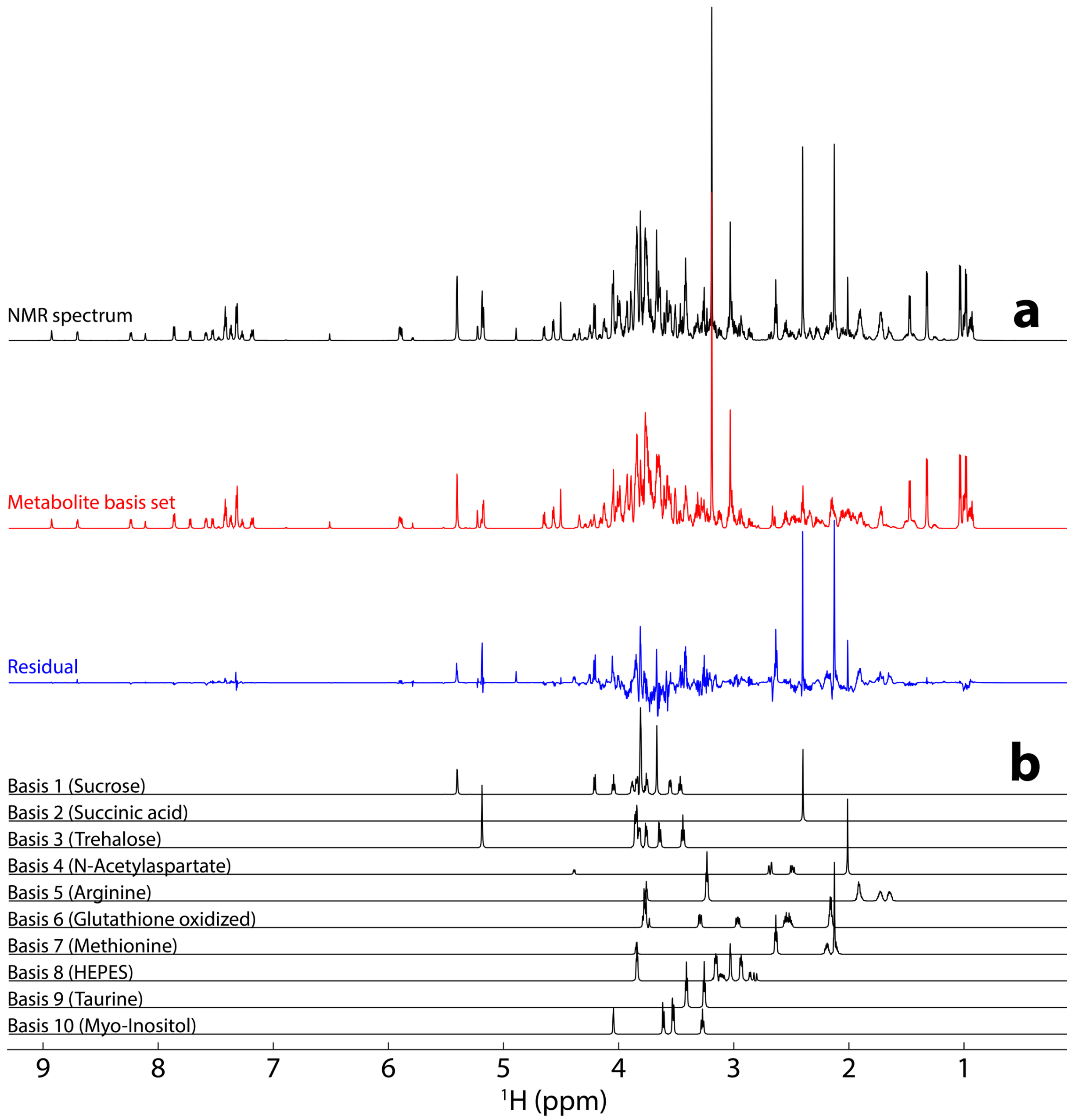

Figure Supplementary 6: a, BATMAN fit of the mBS from our ground-truth set of 53 synthetic metabolite solutions with the first 10 mBS elements excluded from fitting. The NMR spectrum at the top (black) is one of ten ground-truth experimental mixtures for this study, and the same spectrum is shown in Figure 5. The metabolite basis set (mBS) shown in red is the BATMAN fit. The blue trace shows the residuals (wavelet fit) from the Bayesian analysis. **b,** Spectra of the first 10 basis set elements excluded from the fit. In this example, the quantification model accounts for 82% of the total spectral intensity.

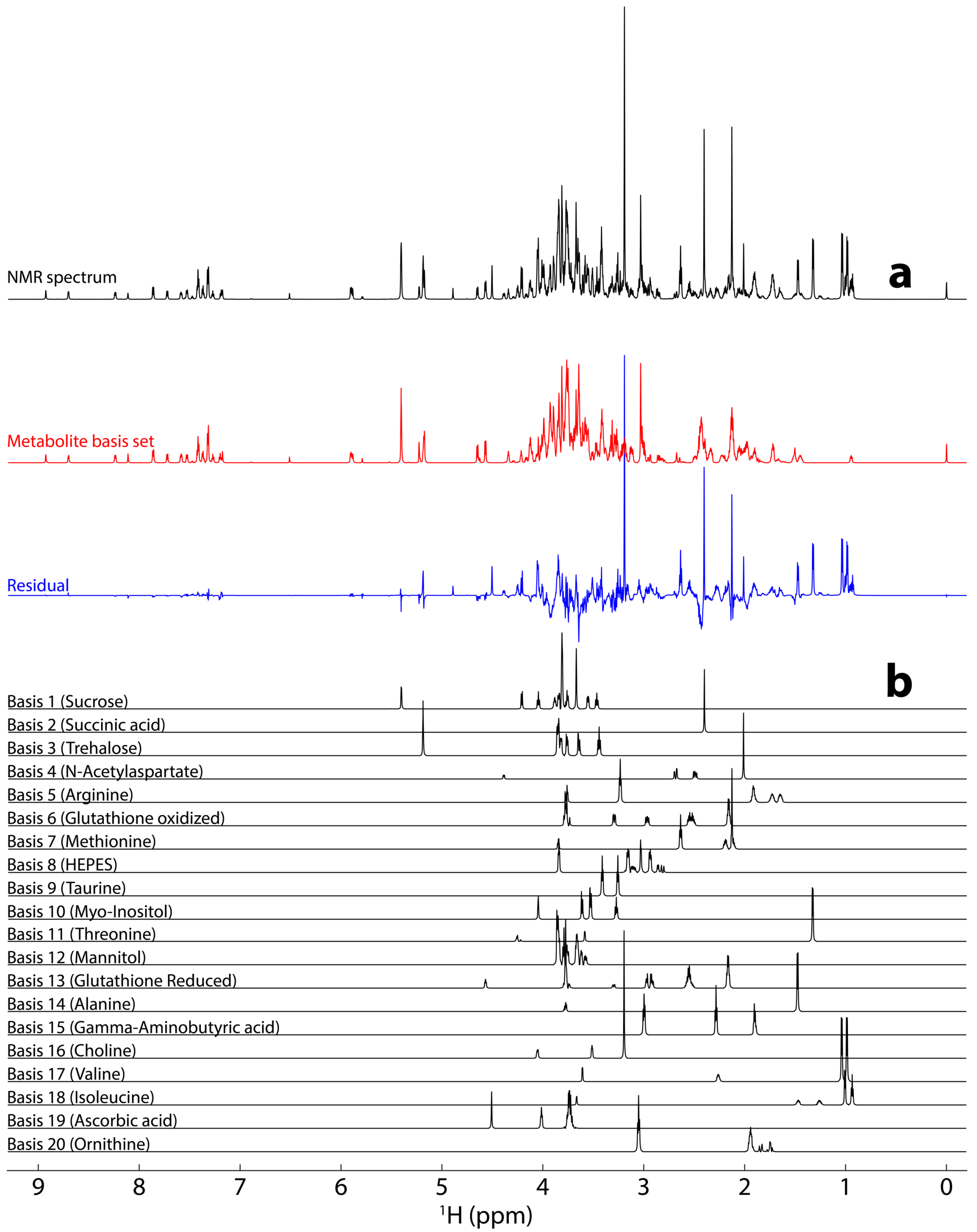

Figure Supplementary 7: a, BATMAN fit of the mBS from our ground-truth set of 53 synthetic metabolite solutions with the first 20 mBS elements excluded from fitting. The NMR spectrum at the top (black) is one of ten ground-truth experimental mixtures for this study, and the same spectrum is shown in Figure 5. The metabolite basis set (mBS) shown in red is the BATMAN fit. The blue trace shows the residuals (wavelet fit) from the Bayesian analysis. **b,** Spectra of the first 20 basis set elements excluded from the fit. In this example, the quantification model accounts for 56% of the total spectral intensity.

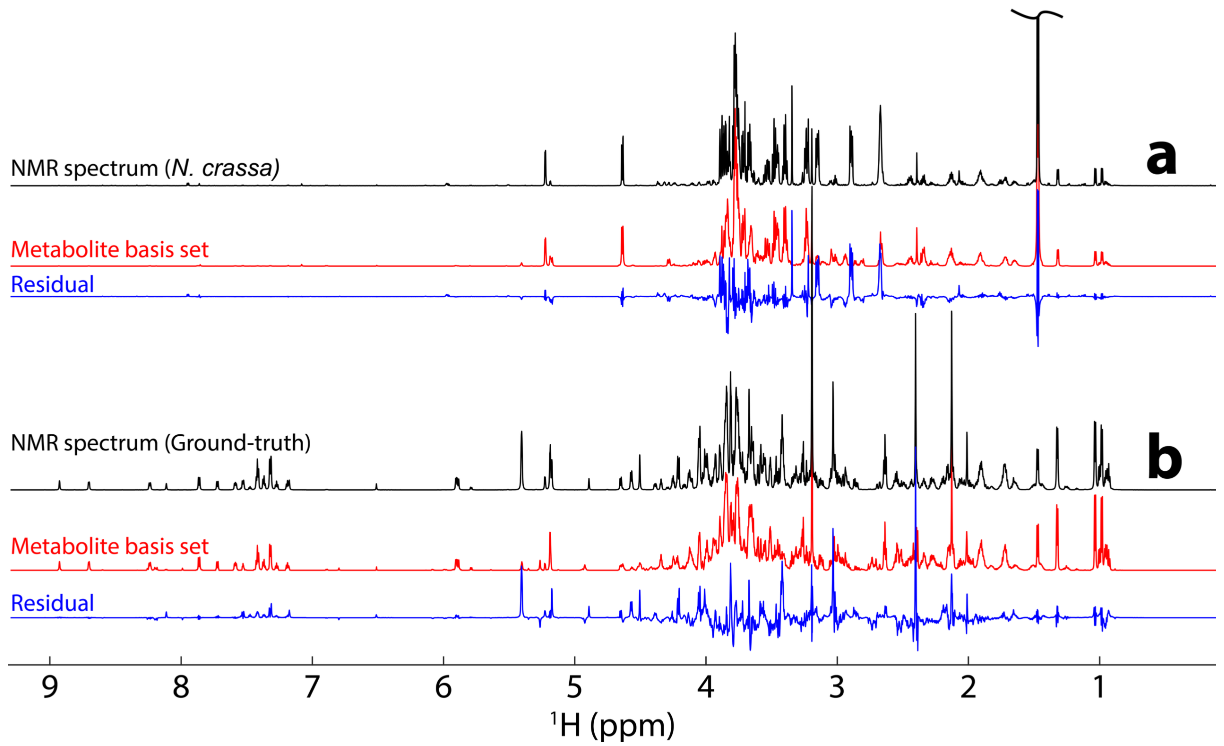

Figure Supplementary 8: a, BATMAN fit of the mBS from the ground-truth mFL to the *N. crassa* unfractionated mixture spectrum. The BATMAN fit explained 83% of the original spectral intensity. **b,** BATMAN fit of the mBS from the *N. crassa* mFL to one of the ground-truth mixture spectra. In this example, the quantification model accounts for 82% of the total spectral intensity.

Ground-Truth Methods

Creating Ground-Truth Spectra

The compounds used to create the single-component ground-truth reference spectra were common metabolites readily available in the laboratory. The milligram amount required for all 53 metabolites was calculated to prepare a 5 mL solution at a 100 mM concentration in H_2O. An approximate amount of powder was weighed for each metabolite and added to a 10 mL glass centrifugal tube. LC/MS grade H_2O was added, the tubes were vortexed, and stored at -20 ˚C. Due to solubility issues, not all metabolites fully dissolved in the solution.

Preparing Fractionation Mixture

10 µL was taken from each single-metabolite reference sample except for aspartate, in which 20 µL was taken and added to a 2 mL screw cap centrifuge tube. The tube was dried in a Centrivap and resuspended in 400 µL of 80:20 LC/MS grade MeOH: H_2O.

HPLC Fractionation

The fraction library was produced by three 100 µL injections using an Agilent 1260 Infinity HPLC with XBridge BEH Amide OBD Prep Column, 130 Å, 5 µm, 10 x 250 mm HILIC column at 25°C. Full scan data was collected using an Agilent Infinity Lab Single Quadrupole MSD in positive ion mode (50-1250 Da). OpenLab ChemStation software was used for data acquisition and visualization.

A 38 min linear gradient of 0.1% formic acid in H_2O (A) and 0.1% formic acid in ACN (B) was used for the fractionation. From 0-20 min, a linear gradient of 5% to 30% A was used, followed by a linear gradient of 30% to 50% A from 20-30 min, all at a flow rate of 3.5 mL/min. From 30-35 min, a linear gradient of 50% to 65% A was used, followed by an isocratic hold from 35-38 min, both at a flow rate of 2 mL/min. A post-time of 8 min was set to allow the system to equilibrate to the initial condition of 5% A before further injections. Between 0.9 and 30 min, 140 equally spaced fractions were collected, approximately 12.5 s per fraction. Fractionation was done over identical fraction vials for all three injections. Between injections 2 and 3 and after injection 3, the vials were dried using a Centrivap. The dried fraction vials were stored at -80 °C before NMR data collection.

Creation of Mixtures for Fitting

Ten mixtures of all metabolites were created. A random number generator was used to generate values between 1-55 µL to decide how much of each single-compound ground-truth sample would be added to each mixture (Table Supplementary 4). The mixtures were then dried using a Centrivap and reconstituted in 550 µL of buffered D_2O (100 mM sodium phosphate buffer and 0.333 mM DSS-D_6 at 7.4 pH). 55 µL of the solutions were pipetted into 1.7 mm Bruker SampleJet NMR tubes.

NMR Data Acquisition

The fractions were reconstituted in 55 µL D_2O buffer and transferred to 1.7 mm Bruker SampleJet tubes. Solvent blanks were placed in positions 1, 96, 97, and 144 amongst the fractions. NMR data were collected using a Bruker Avance Neo console on an Oxford 800 MHz magnet with a 1.7 mm TCI cryoprobe and a cooled SampleJet sample changer. One-dimensional NMR data were acquired at 298K using a “noesypr1d” pulse sequence, and 32,768 points were collected with 8 dummy scans and 64 scans for each sample. The ten mixtures were run on the same instrument. The data were acquired at 298K using a “noesypr1d” pulse sequence, and 32,768 points were collected with 4 dummy scans and 16 scans for each sample. The data were automatically updated to NAN and NMRbox for processing.

Data processing and SAND

Prior to applying SAND, the spectra for ground-truth reference samples and mixtures were processed using the NMRPipe batch processing scheme, and reference deconvolution was applied using DSS as the reference lineshape, with a target linewidth of 1 Hz and a line broadening of 1.5 Hz. The reference samples were then time-domain modeled by SAND over the range of 9.1 ppm to -0.15 ppm.

Relative Concentrations of Basis Set Elements

Because of the limited solubility of some compounds, the expected concentrations do not always match the actual concentrations of the metabolites in the mixtures. To account for this, metabolite concentrations in the mixtures were determined by comparing the integral of isolated resonances to the integral of DSS using Mnova software. The newly calculated concentrations and the BATMAN relative concentrations were then compared in MATLAB.
